# P39 Virtual reality headset as an alternative to Entonox for intraarticular corticosteroid injection

**DOI:** 10.1093/rap/rkac067.039

**Published:** 2022-09-28

**Authors:** Rosie Close, Samantha Small, Olivia Murphy-Parry, Alice Leahy

**Affiliations:** Southampton Children's Hospital, Southampton, United Kingdom; Southampton Children's Hospital, Southampton, United Kingdom; Southampton Children's Hospital, Southampton, United Kingdom; Southampton Children's Hospital, Southampton, United Kingdom

## Abstract

**Introduction/Background:**

Intraarticular corticosteroid injection is an important therapeutic approach in paediatric rheumatology that results in targeted treatment of joint inflammation with minimal systemic side effects. In younger patients general anaesthetic is required but older children are generally able to tolerate the procedure well with Entonox.

Entonox (a mixture of nitrous oxide and oxygen) provides mild analgesia and promotes relaxation. Nitrous oxide is a greenhouse gas, (nearly 300 times more potent than carbon dioxide) and enters the atmosphere after being exhaled by a patient. Therefore, a more environmentally responsible option is required and virtual reality is a potential option.

**Description/Method:**

Virtual Reality (VR) is a computer-generated environment with scenes and objects that appear real, making the participant feel immersed in their surroundings. This environment is perceived through a device known as a Virtual Reality headset or helmet. This simulated experience can be similar to or completely different from the real world.

Applications of virtual reality are expanding and no longer just include entertainment via video games. VR now has many medical applications. After practicing with a virtual reality system for six weeks, people with Parkinson's disease demonstrated improved obstacle negotiation and balance. Oxford VR’s social engagement program designed to help patients overcome anxious social avoidance is now available on the NHS. It applies cognitive behavioural therapy (CBT) techniques within an immersive virtual reality setting.

Virtual reality headsets have been purchased by the play therapy team at Southampton Children’s Hospital. The paediatric rheumatology team was able to utilise one for a 10-year-old boy who required a left knee intraarticular corticosteroid injection. He had the procedure previously with Entonox and although tolerated it was anxious throughout resulting in trepidation at the prospect of a repeat procedure. Consent for a trial with virtual reality headset was obtained as well as consent to video the procedure. An accompanying iPad enabled the health professionals to be able to see the same images as the young person was experiencing enabling them to give relevant comments. The young person remained fully immersed in their virtual reality throughout the procedure and was not able to see what was occurring in ‘real life’. They tolerated the procedure perfectly with no movement of the leg and no verbal reaction. The only noticeable response observed from re-watching the video was the intake of a couple of deep breaths. Immediately after the young person provided verbal feedback: “That was amazing!”

**Discussion/Results:**

Side effects of Entonox are short lasting and include nausea and light-headedness. Some young people cannot tolerate the sensation of feeling out of control with one diabetic patient likening it to experiencing a hypoglycaemic episode and requesting to discontinue.

A study using functional magnetic resonance imaging of healthy patients using VR while exposed to a painful stimulus showed greater than 50% reduction in pain-related brain activity. Cochrane review in 2019 in VR distraction for acute pain in children was non confirmatory and concluded there is a need for larger studies in this area. It is postulated that interacting with immersive VR might divert attention, leading to a slower response to incoming pain signals.

The minimum age limitation for VR gaming is seven but there is no clear consensus on the age recommendation of VR headsets.

Reported side effects of VR include headaches, eye strain, dizziness and nausea. These are triggered by the vergence-accommodation conflict. If a child is susceptible to motion sickness they will likely experience virtual motion sickness.

There was an increase in myopia with the rise of personal handheld devices. As VR screens are very close to the eyes this has raised concern. The American Academy of Ophthalmology state that staring at a VR screen (or any digital device) without breaks may cause eye fatigue due to blinking less often resulting in eye dryness. However, they also comment that although there are no long-term studies there is no reason to be concerned that VR headsets will damage eye development, health or function.

As VR is in its infancy the long-term effects remain unknown. Regular breaks are recommended. As with other digital devices the “20-20-20” rule may be applied: every 20 minutes, adjusting gaze to look at an object at least 20 feet away, for at least 20 seconds.

**Key learning points/Conclusion:**

Due to the environmental impact an alternative to Entonox is urgently required to support young people during invasive procedures. It is important that as many children as possible are able to avoid the risks associated with general anaesthetic.

Virtual reality was very successful in enabling a young person to have a positive experience of a intraarticular corticosteroid injection having previously been anxious about this treatment. The encouraging feedback provided by this patient provides support for continued trial in other patients and also other procedures such as blood tests and subcutaneous medication administration in needle phobic patients.

Although the long-term effects of VR on children are currently unknown the short time of exposure required for accompanying a clinical procedure is unlikely to have a long term impact. However, as always for any new therapeutic intervention, it will be important to continue to monitor the outcomming research with awareness that the VR exposure during medical interventions may not be the only VR experience and very likely not the only digital device the child is exposed to.

The ongoing research into various medical applications including mental health suggests that virtual reality could also become a useful adjunct to paediatric chronic pain management in the future.

